# Purification and Properties of White Muscle Lactate Dehydrogenase from the Anoxia-Tolerant Turtle, the Red-Eared Slider, *Trachemys scripta elegans*

**DOI:** 10.1155/2013/784973

**Published:** 2013-02-21

**Authors:** Neal J. Dawson, Ryan A. V. Bell, Kenneth B. Storey

**Affiliations:** Institute of Biochemistry and Department of Biology, Carleton University, 1125 Colonel By Drive, Ottawa, ON, Canada K1S 5B6

## Abstract

Lactate dehydrogenase (LDH; E.C. 1.1.1.27) is a crucial enzyme involved in energy metabolism in muscle, facilitating the production of ATP via glycolysis during oxygen deprivation by recycling NAD^+^. The present study investigated purified LDH from the muscle of 20 h anoxic and normoxic *T. s. elegans*, and LDH from anoxic muscle showed a significantly lower (47%) *K*_*m*_ for L-lactate and a higher *V*_max_ value than the normoxic form. Several lines of evidence indicated that LDH was converted to a low phosphate form under anoxia: (a) stimulation of endogenously present protein phosphatases decreased the *K*_*m*_ of L-lactate of control LDH to anoxic levels, whereas (b) stimulation of kinases increased the *K*_*m*_ of L-lactate of anoxic LDH to normoxic levels, and (c) dot blot analysis shows significantly less serine (78%) and threonine (58%) phosphorylation in anoxic muscle LDH as compared to normoxic LDH. The physiological consequence of anoxia-induced LDH dephosphorylation appears to be an increase in LDH activity to promote the reduction of pyruvate in muscle tissue, converting the glycolytic end product to lactate to maintain a prolonged glycolytic flux under energy-stressed anoxic conditions.

## 1. Introduction

Lactate dehydrogenase (LDH; E.C. 1.1.1.27) is a critical enzyme involved in anaerobic metabolism. LDH catalyzes the following reversible reaction:

(1) $$\begin{array}{c} {\text{NAD}}^{+}+\text{L-lactate}⟷\text{NADH}+{\text{H}}^{+}+\text{pyruvate}. \end{array}$$

In this capacity, LDH favors the pyruvate reducing direction in skeletal muscle tissue, converting the glycolytic end product to lactate and regenerating the NAD^+^ pools to maintain a prolonged glycolytic flux [1]. This process is especially critical to those organisms that enter periodically into hypoxic/anoxic environments, where maintaining NAD^+^/NADH balance is essential for ATP production.

Under low oxygen insult, organisms often rely solely on the glycolytic pathway to produce ATP. The greatly reduced production of ATP via glycolysis, as compared to that of oxidative phosphorylation, results in difficult challenges for anoxia-tolerant organisms to overcome. Several of these organisms employ alternate anaerobic pathways to increase ATP yield and/or depress their metabolic rate to survive the low oxygen stress [2]. Furthermore, these organisms typically need to safeguard against the accumulation of acidic glycolytic end products such as lactate, which disrupts cellular homeostasis throughout prolonged exposure to anoxia [2]. 

Freshwater turtles, *Trachemys scripta elegans*, have demonstrated a remarkable ability to survive submerged in cold water for 4-5 months during the winter to escape freezing air temperatures. While submerged, these turtles can absorb sufficient O_2_ to drive their metabolic needs [3]; however, as oxygen levels drop in ice-locked lakes and rivers, these turtles become facultative anaerobes. *T. s. elegans* employ several key methods of surviving these harsh conditions including: (a) suppression of their metabolic rate to 10–20% of the aerobic rate [2], (b) a complete switch to glycolysis for ATP production [2], and (c) buffering against severe acidosis through the use of unique methods for storing lactate in their shells [4].

Reversible protein phosphorylation continues to emerge as an increasingly common method of posttranslationally modifying and regulating enzymes within anoxia-tolerant animals. Phosphorylation has been found to be critical in regulating carbohydrate catabolism [5], amino acid metabolism [6, 7], ATPase functioning [8], antioxidant defense [9], and many other processes, and is considered critical to low-oxygen survival. Phosphorylation of LDH has been observed in a number of earlier studies [10, 11], with recent work indicating that LDH from the anoxia-tolerant turtle liver is regulated by reversible phosphorylation [12]. The present study investigates the physical, kinetic, and regulatory properties of turtle muscle LDH and presents a role for reversible phosphorylation as the main form of regulating LDH in response to anoxia insult.

## 2. Materials and Methods

### 2.1. Experimental Animals and Tissue Sampling

Adult *T. s. elegans* is obtained during the winter from Wards Natural Science, Mississauga, ON, Canada. Turtles, weighing between 850 and 1800 grams, were housed in deep tanks containing dechlorinated water at 7°C, a small platform, and were fed trout pellets, lettuce, and egg shells. Half the turtles (~5) were sampled directly from the tanks to comprise the control (normoxic) group. The remaining turtles (~5) were sealed in the tanks, and the tanks were bubbled with 100% nitrogen gas at 4°C for 20 h. Wire mesh was placed below the surface of the water to mimic ice coverage, ensuring that no turtle could surface during the induced anoxic exposure. For sampling, animals were killed by severing the head, and then white muscle from the neck retractor was quickly harvested, immediately frozen in liquid nitrogen, and stored at −80°C (a protocol approved by the University Animal Care Committee and meeting the guidelines of the Canadian Council on Animal Care).

 Chemicals, biochemicals, chromatography media, and coupling enzymes were from Sigma Chemical Co. (St. Louis, MO, USA), and ProQ-Diamond Phosphoprotein stain was from Invitrogen (Eugene, OR, USA). Primary antibodies to SUMO 1 and SUMO 2/3 were graciously gifted by the Hallenbeck lab (Clinical Investigations Section Stroke Branch, NINDS, Bethesda, MD, USA).

### 2.2. Preparation of Tissue Extracts

Samples of frozen white muscle were homogenized 1 : 5 w : v in ice-cold buffer A: 20 mM potassium phosphate (KPi) buffer, pH 7.2 containing 15 mM *β*-glycerophosphate, 1 mM EGTA, 1 mM EDTA, 15 mM *β*-mercaptoethanol, and 1 mM phenylmethylsulfonyl fluoride (PMSF). Turtle muscle homogenates were centrifuged at 13 500 ×g at 4°C, the supernatant was decanted, and both the supernatant and the pellet were held on ice until use.

### 2.3. Purification of LDH

Muscle extracts were prepared (1 : 5 w : v) in homogenization buffer A. A 3 mL aliquot of crude extract was applied to a Cibacron Blue 3GA column (1.5 cm × 10 cm) equilibrated in buffer A, washed with 30 mL of buffer to remove unbound protein, and then eluted with a linear KCl gradient (0–2 M in 40 mL) in the same buffer. Fractions of 0.8 mL were collected, and 5 uL from each fraction was assayed. The peak fractions from the Cibacron Blue 3GA column were pooled, diluted 20 times in buffer A, and then applied to an oxamate column (1.5 cm × 5 cm), equilibrated in the same buffer. The column was eluted in the same fashion as the Cibacron Blue 3GA column, and peak fractions were pooled. The purity of LDH was checked by running samples on SDS-PAGE (described in the following) with Coomassie blue staining.

### 2.4. LDH Enzyme Assay

LDH activity was measured as the rate of consumption or production of NADH. Optimal assay conditions for LDH in the lactate-oxidizing direction were 100 mM KPi buffer pH 7.2, 80 mM L-lactate, and 2 mM NAD^+^, in a total volume of 200 *μ*L with 30 *μ*L of purified enzyme extract per assay. Enzyme activity was assayed with a Thermo Labsystems Multiskan spectrophotometer at 340 nm. One unit of LDH activity in the lactate-oxidizing direction is defined as the amount of enzyme that produced 1 *μ*mol of NADH per minute at 25°C. 

Optimal conditions for LDH in the pyruvate-reducing direction were 100 mM KPi buffer pH 7.2, 2 mM pyruvate, and 0.2 mM NADH, in a total volume of 200 *μ*L with 10 *μ*L of purified enzyme extract per assay. Enzyme activity was assayed with a Thermo Labsystems Multiskan spectrophotometer at 340 nm. One unit of LDH activity in the pyruvate-reducing direction is defined as the amount of enzyme that consumed 1 *μ*mol of NADH per minute at 25°C.

Data were analyzed using the Kinetics v.3.5.1 program [13]. Protein concentrations were determined using the Coomassie blue dye-binding method with the BioRad prepared reagent and bovine serum albumin as the standard.

### 2.5. *In Vitro* Incubation to Stimulate Protein Kinases and Phosphatases

 Samples of muscle extracts, prepared as previously described in buffer A, were filtered through a G50 spun column equilibrated in buffer B (20 mM KPi, 10% v : v glycerol, 15 mM *β*-mercaptoethanol, and pH 7.2). Aliquots of the filtered supernatants were incubated overnight at 4°C with specific inhibitors and stimulators of protein kinases and phosphatases, as described in MacDonald and Storey [14]. Each aliquot was mixed 1 : 2 v : v with the appropriate solutions in buffer C that were designed to stimulate either protein kinases or phosphatases.

- Stop conditions: 2.5 mM EGTA, 2.5 mM EDTA, and 30 mM *β*-glycerophosphate.
- Stimulation of endogenous kinases 5 mM Mg-ATP, 30 mM *β*-glycerophosphate, and one of the following:
  1. 1 mM AMP, to stimulate AMP-activated protein kinase (AMPK);
  2. 1.3 mM CaCl_2_ and 7 *μ*g/mL phorbol myristate acetate (PMA), to stimulate protein kinase C (PKC);
  3. 1 mM cAMP, to stimulate protein kinase A (PKA);
  4. 1 mM cGMP, to stimulate protein kinase G (PKG);
  5. 1 U of calf intestine calmodulin and 1.3 mM CaCl_2_, to stimulate calcium-calmodulin protein kinase (CaMK);
- Stimulation of endogenous phosphatases: 5 mM CaCl_2_ and 5 mM MgCl_2_.

After incubation, low molecular weight metabolites and ions were removed from the extracts by centrifugation for 2 min at 2000 rpm through small spun columns of Sephadex G-50 equilibrated in buffer A, and the *K*_*m*_ of lactate was reassessed for each condition.

### 2.6. Dot Blotting Analysis of Purified LDH

Control and anoxic white muscle samples were purified as previously outlined (Figure 2). Soluble protein concentration was measured by the Coomassie blue dye-binding method. Samples were applied to nitrocellulose membranes using a Bio-Dot microfiltration apparatus (Bio-Rad, Hercules, CA, USA) using the following protocol.

1. Nitrocellulose membrane was cut to match the size of the Bio-Dot microfiltration apparatus and was prewetted in Tris-buffered saline (TBS) (100 mM Tris, 1.4 M NaCl, pH 7.6).
2. 100 *μ*L of purified control and anoxic LDH was applied using the Bio-Dot microfiltration apparatus and allowed to filter through the membrane via gravity flow for 1 h.
3. When the protein sample had filtered through completely, the membranes were washed twice with 200 *μ*L TBS using gentle vacuum suction.
4. The membrane was then removed from the apparatus, placed in a container, and washed in Tris-buffered saline containing 0.05% Triton-X (TBST) 3 times for 5 minutes.
5. The membrane was cut, separating 4 control and 4 anoxic samples in each membrane strip, and placed into a separate container.
6. The strips were then blocked with 5 mL of 1 mg/mL (70–100 kDa) PVA in TBST for 45 seconds then washed 3 times in TBST for 5 minutes.
7. Membranes were then incubated with primary antibody in 10 mL of TBST overnight at 4°C recognizing each of the following posttranslational modifications:
  - *serine phosphorylation* (antiphosphoserine (618100), anti-rabbit, Invitrogen, Carlsbad, CA, USA);
  - *threonine phosphorylation* (antiphosphothreonine (718200), anti-rabbit, Invitrogen, Carlsbad, CA, USA);
  - *tyrosine phosphorylation* (anti-phosphotyrosine (136600), anti-mouse, Invitrogen, Carlsbad, CA, USA);
  - *acetylation* (pan-acetyl (C4)-R (sc-8663-R), anti-rabbit, Santa Cruz Biotechnology, Santa Cruz, CA, USA);
  - *ubiquitination* (antiubiquitin (ab19247), anti-rabbit, abcam, Cambridge, UK);
  - *methyl-lysine phosphorylation* (antimethylated lysine (SPC-158F), anti-rabbit, StressMarq, Biosciences Inc., Victoria, BC, Canada);
  - *nitrosylation* (antinitrosocysteine (ab50185), anti-rabbit, abcam, Cambridge, UK);
  - *SUMOylation 1* (anti-SUMO 1, anti-rabbit, Clinical Investigations Section Stroke Branch, NINDS, Bethesda, MD, USA);
  - *SUMOylation 2/3* (anti-SUMO 2/3, anti-rabbit, Clinical Investigations Section Stroke Branch, NINDS, Bethesda, MD, USA);
8. After washing with TBST (3 times for 5 minutes), membranes were incubated with anti-rabbit IgG secondary antibody (1 : 1000 dilution) for 1 h and washed three times in TBST (5 min).
9. Immunoreactive dots were visualized by enhanced chemiluminescence on the ChemiGenius Bioimaging System (Syngene, Frederick, MD, USA).
10. Dot intensities were quantified using GeneTools software.
11. Coomassie blue staining was used to confirm equal loading of sample and to standardize immunoblotting dot intensities.

### 2.7. Statistical Analyses

 The mean values for all kinetic parameters measured were compared between control and anoxic conditions using the Student's *t*-test (2 tails, unequal variance). The same analysis was done for all dot blots, with relative dot intensities being compared between the two conditions. Incubations, modifying LDH phosphorylation state, were analyzed using an ANOVA followed by a post hoc Dunnett's test (*P* < 0.05) to assess changes in kinetic values.

## 3. Results and Discussion

### 3.1. LDH Purification and Kinetics

**Figure 1:**
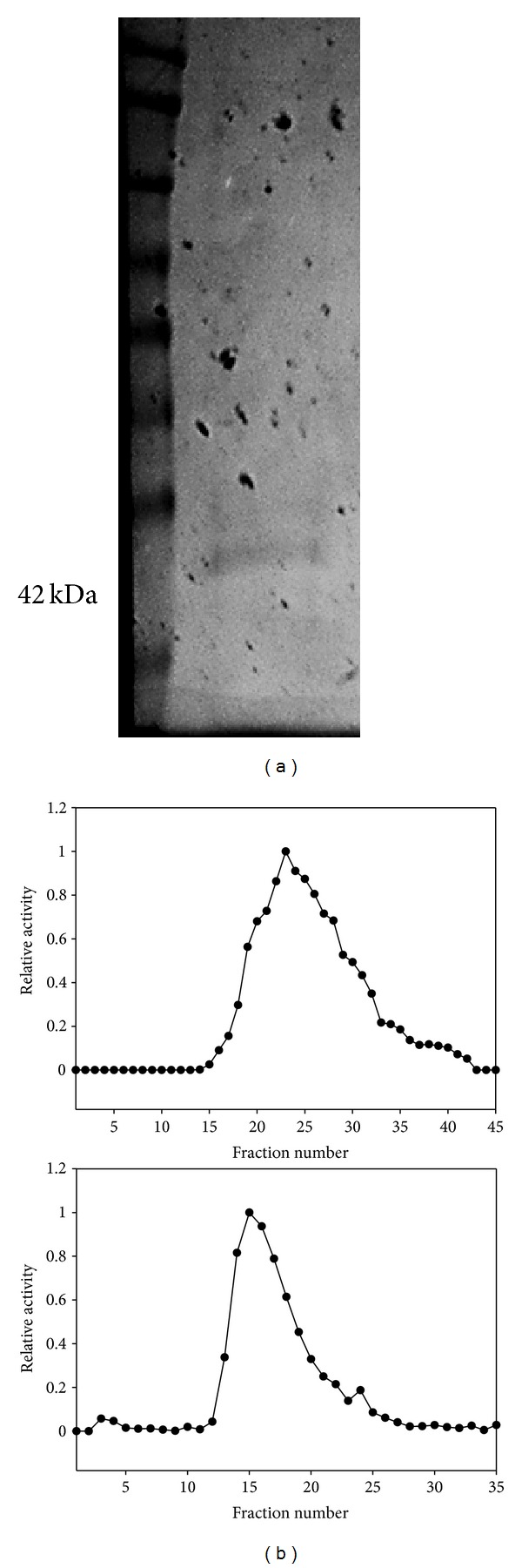
Purified normoxic *T. s. elegans* white muscle LDH. (a) Silver-stained gel showing the FroggaBio protein ladder (left) and purified control white muscle LDH. (b) Example elution profiles of control LDH from blue agarose (top) and oxamate (bottom) columns.

**Figure 2:**
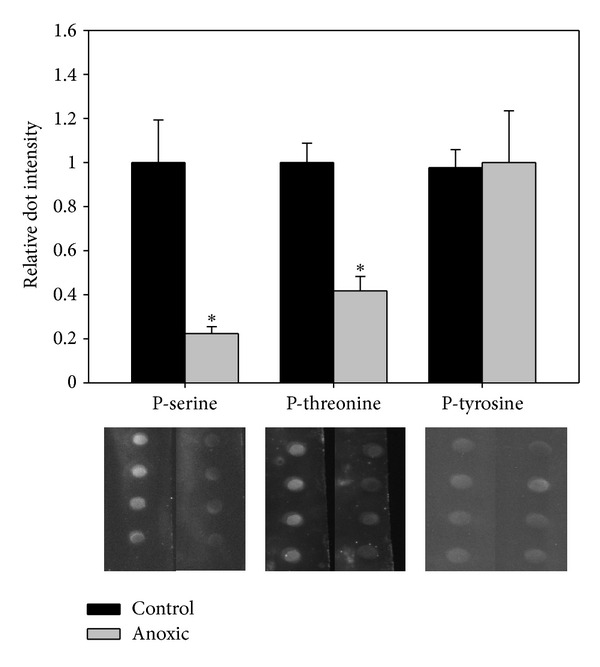
Analysis of normoxic and anoxic white muscle LDH phosphorylation state through dot blots. Chemiluminescent dots are shown below the corresponding bars. Data are means ± SEM, *n* = 4 independent determinations. *Significantly different from the corresponding control value using the Student's *t*-test, *P* < 0.05.

LDH from the white muscle of the turtle was purified to electrophoretic homogeneity through the use of two affinity columns: blue agarose and N(6-aminohexyl) oxamate (Figure 1). The initial purification step using blue agarose only resulted in a 1.1-fold purification of normoxic LDH, however oxamate agarose removed all other proteins leaving normoxic turtle muscle LDH electrophoretically pure with an overall yield of 23% (Table 1). Anoxic turtle muscle LDH was also purified using blueagarose and oxamate agarose under the same conditions as normoxic LDH (See Supplementary Table 1 in Supplementary Material available online at http://dx.doi.org/10.1155/2013/784973). LDH activity eluted in a single peak for both the blue-agarose and oxamate columns and typical elution profiles from both affinity columns are shown in Figure 1. Both fold purifications and percent yields obtained through the use of these two columns are very similar to an identical purification process utilized for *T. s. elegans* liver LDH [12]. The yield obtained from the purification scheme used here is also in line with other purifications within the muscle of an unrelated animal, the lizard, *Agama stellio stellio* [15].

Kinetic analyses of purified skeletal muscle LDH from control and anoxic conditions revealed distinct differences for this enzyme between the two conditions. For instance, the *K*_*m*_ of lactate for 20 h anoxic LDH was approximately 50% of that seen for LDH from the control condition. Furthermore, the *V*_max⁡_ for anoxic LDH was nearly 4-fold higher in the lactate-oxidizing direction and over 7-fold higher in the pyruvate-reducing direction, as compared to LDH from the control condition (Table 2; Supplementary Figure 1). These results suggest that turtle muscle LDH may function more efficiently during anoxia. Given that muscle cells are typically nongluconeogenic and anaerobic glycolysis is the sole means of energy production during anoxia, LDH activation may be necessary to maintain NAD^+^ levels and prevent the accumulation of pyruvate within muscle cells. Supporting this finding is the fact that previous studies with anoxia-tolerant turtles show that most tissues, including skeletal muscle, show a dramatic increase in tissue lactate concentration during anoxia [16]. The lactate produced can also be exported into the blood and distributed throughout the body, including the shell, which takes up a significant portion of tissue-born lactate [17]. 

### 3.2. LDH Phosphorylation State

**Figure 3:**
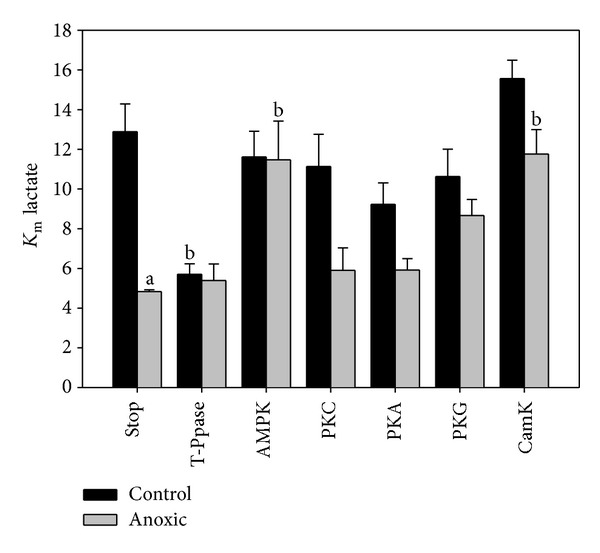
Effect of incubations that stimulated endogenous protein kinases and phosphatases on *T. s. elegans* white muscle LDH K_m_ lactate. The Stop condition indicates that both protein kinases and phosphatases were inhibited. Data are means ± SEM, *n* = 3 independent determinations. (a) Significantly different from the control Stop condition using the Student's *t*-test, *P* < 0.05. (b) Significantly different from the corresponding value from the Stop condition using the Dunnett's test, *P* < 0.05.

Although LDH is generally suited to function under anaerobic conditions, it is the extent and duration of the oxygen deprivation for *T. s. elegans *that suggests the need to further regulate this enzyme during long-term anoxia exposures. The kinetic changes outlined above are evidence that this may be the case, and the most common posttranslational form of regulation is reversible protein phosphorylation. The phosphorylation state of LDH was assessed through the use of dot blots and phosphospecific antibodies. Dot-blot analysis of LDH using phospho-serine and -threonine antibodies demonstrates that anoxic LDH shows significantly less serine (78%) and threonine (58%) phosphorylation as compared to control (Figure 3). LDH has been found to be phosphorylated in a number of other studies, including the in liver of *T. s. elegans* [12]. However, in that study the anoxic form of LDH was found to be only slightly more phosphorylated as compared to the control enzyme. This difference may reflect the need for tissue-specific differences in LDH regulation based on the varying role of each tissue during hypometabolism. Consistent among these two studies is that the increase in bound phosphate on LDH in either liver or muscle tissue decreased LDH activity. Other studies have also shown that LDH can be phosphorylated on serine/threonine residues and that some of these changes may be important to its role in cell metabolism [18]. Furthermore, tyrosine residue phosphorylation on LDH has also been shown in several studies [10, 11], with some indication that tyrosine phosphorylation has significant kinetic consequences [19]. LDH tyrosine phosphorylation was investigated in this study; however, there was no difference in the level of this posttranslational modification between the two conditions, suggesting that a novel phosphorylation event may be responsible for the observed kinetic differences (Figure 3). 

To assess the effect of changes in phosphorylation state on the kinetic characteristics of both control and anoxic LDH, crude preparations were incubated in conditions that would stimulate protein kinases or phosphatases and the *K*_*m*_ of lactate was reassessed. Under conditions that inhibited both kinases and phosphatases (STOP condition) a similar statistically significant difference in the control and anoxic *K*_*m*_ of lactate was seen for these crude preparations as was observed for purified samples (compare Figure 3 and Table 2). Incubations that stimulated many endogenous protein phosphatases caused a significant decrease in the *K*_*m*_ of lactate for control LDH; however, anoxic LDH remained unaffected. Subsequently, incubations that investigated the role of specific protein kinases in altering LDH kinetics identified AMP-activated protein kinase (AMPK) and calcium/calmodulin protein kinase (CamK) as kinases that caused an approximately 2-fold increase in the *K*_*m*_ of lactate for anoxic LDH. Both of these kinases have known roles in metabolism and may potentially be important regulators of LDH *in vivo*. Studies by Yasykova and colleagues [18] coincide with the present study in that CamK was identified as a kinase that acted upon LDH; however, its action was linked to significant increases in LDH activity which is counter to that seen in this study. While no study has identified AMPK as a potential modifier of LDH prior to this study, a search of phosphorylatable motifs within the *T. s. elegans* LDH protein sequence using Scansite (http://scansite.mit.edu/) revealed that, under the lowest stringency, AMPK has a known motif within LDH (*T.s. elegans *LDH sequence found in the appendix). Scansite also indicated that there are numerous other protein kinases that may be effective in phosphorylating LDH including various PKC isoforms as well as PKA, both of which had a negligible effect on LDH kinetics in this study.

### 3.3. Additional LDH Posttranslational Modifications

**Figure 4:**
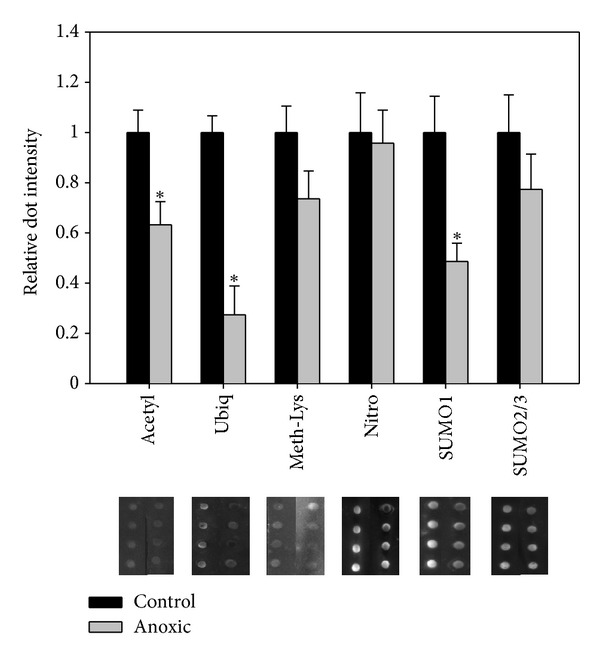
Posttranslational modification of purified *T. s. elegans* white muscle LDH assessed using dot blots. Chemiluminescent dots are shown below their corresponding dots. Data are means ± SEM, *n* = 4 independent determinations. *Significantly different from the corresponding control value using the Student's *t*-test, *P* < 0.05.

In addition to phosphorylation, the presence of numerous other posttranslational modifications on purified control and anoxic LDH was assessed through dot blots.  Figure 4 indicates that anoxic LDH was 37%, 73%, and 51% less acetylated, ubiquitinated, and SUMOylated (SUMO1), respectively, as compared to control LDH. Significant changes in acetylation levels for LDH were recently observed for *T. s. elegans *liver LDH; however, similar to the phosphorylation state, LDH from the anoxic animal was significantly more acetylated as compared to control LDH [12]. This is clearly opposite to that seen in this study and again is evidence of tissue-specific adaptations to anoxia within the red-eared slider. The role of acetylation within anoxia-tolerant turtles is not known; however, one study has suggested that it may confer some low temperature stability by decreasing the number of charged residues present on the surface of the protein [20]. Further studies are needed to assess if there is a structural stability difference between control and anoxic LDH and whether this is related to the difference in acetylation levels.

Similar to acetylation, the addition of ubiquitin or ubiquitin-like molecules (SUMO protein) to turtle LDH has unknown effects on enzyme function. Classically, ubiquitination of cellular proteins leads to proteasome-mediated degradation. Previous studies have shown that muscle LDH ubiquitination may be sensitive to the oxidative stress, with increased hydrogen peroxide-derived free radicals leading to greater ubiquitination and degradation [21]. This may be reflected in this study with the population of LDH from the anoxic turtle muscle being significantly less ubiquitinated as compared to normoxic LDH. This may allow for LDH to remain at appropriate levels within the muscle during anoxia and facilitate the clearance of pyruvate, which is necessary for anaerobic glycolysis to proceed. 

Although similar to ubiquitin in structure, the function of SUMO protein conjugates within the cell is much less understood. Interestingly, one study has shown that posttranslational SUMOylation is very sensitive to cell stress, with dramatic changes in SUMOylation occurring with oxidative or alkylation stress [22]. The same study identified muscle LDH as one of the few enzymes of intermediary metabolism that was SUMOylated. While the consequence of LDH SUMOylation is unknown, research over the past decade has identified posttranslational modification as being an important regulator of enzyme activity, protein-protein interactions, and protein stability [23]. Further investigation is required to assess the role of turtle muscle LDH as, the kinetic changes observed between control and anoxic LDH seem mainly based on changes in phosphorylation state. 

In addition to the above-mentioned posttranslational modifications, levels of methylation (lysines), nitrosylation, and SUMOylation (SUMO2/3) were investigated as potentially important regulatory modifications for LDH during turtle anoxia. Our results indicate that neither of these modifications changed significantly between control and stress conditions, suggesting that they are unlikely to be key regulatory mechanisms during anoxia.

## 4. Conclusion

 The kinetic alterations identified in this study suggest that white muscle LDH from normoxic and anoxic *T. s. elegans* exists in two distinct forms, with control muscle LDH being a more highly phosphorylated form in comparison to anoxic muscle LDH. Incubations that stimulated protein kinases and phosphatases indicated that the kinetic changes were due to the reversible phosphorylation of LDH, with dephosphorylation resulting in a more active muscle LDH during anoxia and likely an increased recycling of NAD^+^ during this period. This correlates well with the increased necessity for the maintenance of glycolytic flux during anoxia to sustain the essential cellular functions. Additionally, this study identified numerous other LDH posttranslational modifications (acetylation, ubiquitination, and SUMOylation) that changed between normoxic and anoxic states, possibly suggesting further regulatory mechanisms for nonkinetic functions.

## Supplementary Material

The supplementary material contains a table that outlines the purification scheme and typical yield for white muscle LDH from the anoxia-tolerant *T. s. elegans*. The results closely resemble those shown for control *T. s. elegans* within the article (Table 1). Additionally, the supplementary material contains a representative kinetic plot showing the activity of control and anoxic LDH with respect to changing lactate concentrations. This figure further demonstrates the dramatic increase in anoxic LDH Vmax as well as the decrease in anoxic LDH Km lactate.

## Figures and Tables

**Table 1:** Purification scheme for *T.  s. elegans* normoxic white muscle LDH.

Purification step	Total protein (mg)	Total activity (U)	Specific activity (U/mg)	Fold purification	% Yield
Crude	25	16	0.64	—	—
Blue agarose	17	12	0.71	1.1	75
Oxamate	0.16	3.8	23	32	23

**Table 2:** Control and 20 h anoxic *T. s. elegans *purified skeletal muscle LDH kinetics.

	Control	20 h anoxic
*K*_*m*_ lactate (mM)	17 ± 2	8 ± 1*
*K*_*m*_ NAD^+^ (mM)	0.38 ± 0.06	0.5 ± 0.1
*V*_max⁡_ (U/mg)	0.19 ± 0.01	0.75 ± 0.02*

*K*_*m*_ pyruvate (mM)	0.15 ± 0.03	0.140 ± 0.003
*V*_max⁡_ (U/mg)	0.52 ± 0.03	3.8 ± 0.2*

Data are means ± SEM, *n* = 4 individual determinations. *K*_*m*_ values were determined at optimal cosubstrate concentrations (defined in Materials and Methods). *Significantly different from the corresponding control value using a two-tailed Student's *t*-test, *P* < 0.05.

## Appendix

### *T. s. elegans *LDH Amino Acid Sequence

1 msvkelliqnvhkeehshahnkitvvgvgavgmacaisilmkdladelalvdviedklrg;
61 emldlqhgslflrtpkivsgkdysvtahsklviitagarqqegesrlnlvqrnvnifkfi;
121 ipnvvkhspdctllvvsnpvdiltyvawkisgfpkhrvigsgcnldsarfrylmggklgi;
181 hslschgwiigehgdssvpvwsgvnvagvslkalypdlgtdadkehwkevhkqvvdsaye;
241 viklkgytswaiglsvadlaetimrnlrrvhpistmvkgmygihddvflsvpcvlgysgi;
301 tdvvkmtlkseeeeklrksadtlwgiqkelqf.
