# Supplementary material for: Purification and Properties of White Muscle Lactate Dehydrogenase from the Anoxia-Tolerant Turtle, the Red-Eared Slider, Trachemys scripta elegans
Source: Enzyme Res. 2013 Feb 21;2013:784973. doi: 10.1155/2013/784973 (PMC3594981; doi:10.1155/2013/784973)
Supplement: Supplementary file 1 — The supplementary material contains a table that outlines the purification scheme and typical yield for white muscle LDH from the anoxia-tolerant T. s. elegans. The results closely resemble those shown for control T. s. elegans within the article (Table 1). Additionally, the supplementary material contains a representative kinetic plot showing the activity of control and anoxic LDH with respect to changing lactate concentrations. This figure further demonstrates the dramatic increase in anoxic LDH Vmax as well as the decrease in anoxic LDH Km lactate. [file 784973.f1.doc]

| Purification Step | Total Protein (mg) | Total Activity  (U) | Specific Activity (U/mg) | Fold Purification | % Yield |
| --- | --- | --- | --- | --- | --- |
| Crude | 27 | 15 | 0.56 | - | - |
| Blue Agarose | 15.7 | 12 | 0.76 | 1.4 | 79 |
| Oxamate | 0.18 | 7.5 | 41 | 74 | 50 |

**Supplementary Table:** Purification scheme for *T.s. elegans* anoxic white muscle LDH.

**Supplementary Figure:** Representative kinetic plot showing the activity of control and anoxic LDH with respect to changing lactate concentratons.
